# A Practical Guide to Small Protein Discovery and Characterization Using Mass Spectrometry

**DOI:** 10.1128/jb.00353-21

**Published:** 2022-01-18

**Authors:** Christian H. Ahrens, Joseph T. Wade, Matthew M. Champion, Julian D. Langer

**Affiliations:** a Agroscope, Method Development and Analytics & SIB Swiss Institute of Bioinformatics, Wädenswil, Switzerland; b Wadsworth Center, New York State Department of Health, Albany, New York, USA; c Department of Biomedical Sciences, School of Public Health, University at Albany, Albany, New York, USA; d Department of Chemistry and Biochemistry, University of Notre Dame, Notre Dame, Indiana, USA; e Mass Spectrometry and Proteomics, Max Planck Institute of Biophysics, Frankfurt am Main, Germany; f Proteomics, Max Planck Institute for Brain Research, Frankfurt am Main, Germany; The Ohio State University

**Keywords:** proteomics, small protein, sproteins, SEP, microprotein, genome annotation, LC-MS/MS, shotgun proteomics, top-down proteomics, sample preparation

## Abstract

Small proteins of up to ∼50 amino acids are an abundant class of biomolecules across all domains of life. Yet due to the challenges inherent in their size, they are often missed in genome annotations, and are difficult to identify and characterize using standard experimental approaches. Consequently, we still know few small proteins even in well-studied prokaryotic model organisms. Mass spectrometry (MS) has great potential for the discovery, validation, and functional characterization of small proteins. However, standard MS approaches are poorly suited to the identification of both known and novel small proteins due to limitations at each step of a typical proteomics workflow, i.e., sample preparation, protease digestion, liquid chromatography, MS data acquisition, and data analysis. Here, we outline the major MS-based workflows and bioinformatic pipelines used for small protein discovery and validation. Special emphasis is placed on highlighting the adjustments required to improve detection and data quality for small proteins. We discuss both the unbiased detection of small proteins and the targeted analysis of small proteins of interest. Finally, we provide guidelines to prioritize novel small proteins, and an outlook on methods with particular potential to further improve comprehensive discovery and characterization of small proteins.

## INTRODUCTION

Large-scale discovery of small proteins (<∼50 amino acids; also referred to as “sproteins,” “short ORF-encoded proteins” (SEPs), or “microproteins”) has relied primarily on computational analysis of genome sequences (1), and genome-scale experimental measurements of transcription and translation such as transcriptome sequencing (RNA-seq) (2) and ribosome profiling (Ribo-seq) (3, 4). Computational analyses can leverage the thousands of available genome sequences, but still require experimental support to serve as conclusive evidence. The major experimental approaches used to date are genome-scale analyses of transcription and translation. These methods share the advantage of amplified RNA-based detection (allowing sensitivity down to single molecules), high data acquisition speeds, and—with appropriate modification as differential RNA-seq (dRNA-seq) (5) or Ribo-RET (6)—the ability to identify transcription and translation initiation sites and potentially even very low-abundance proteins. However, all RNA-based approaches only provide indirect evidence of small protein expression, and some of the translation products detected by Ribo-seq may be unstable. Moreover, RNA-based approaches cannot provide data on post-translational modifications, other processing steps like signal peptide cleavage, or maturation that proteins can undergo.

**FIG 1:**
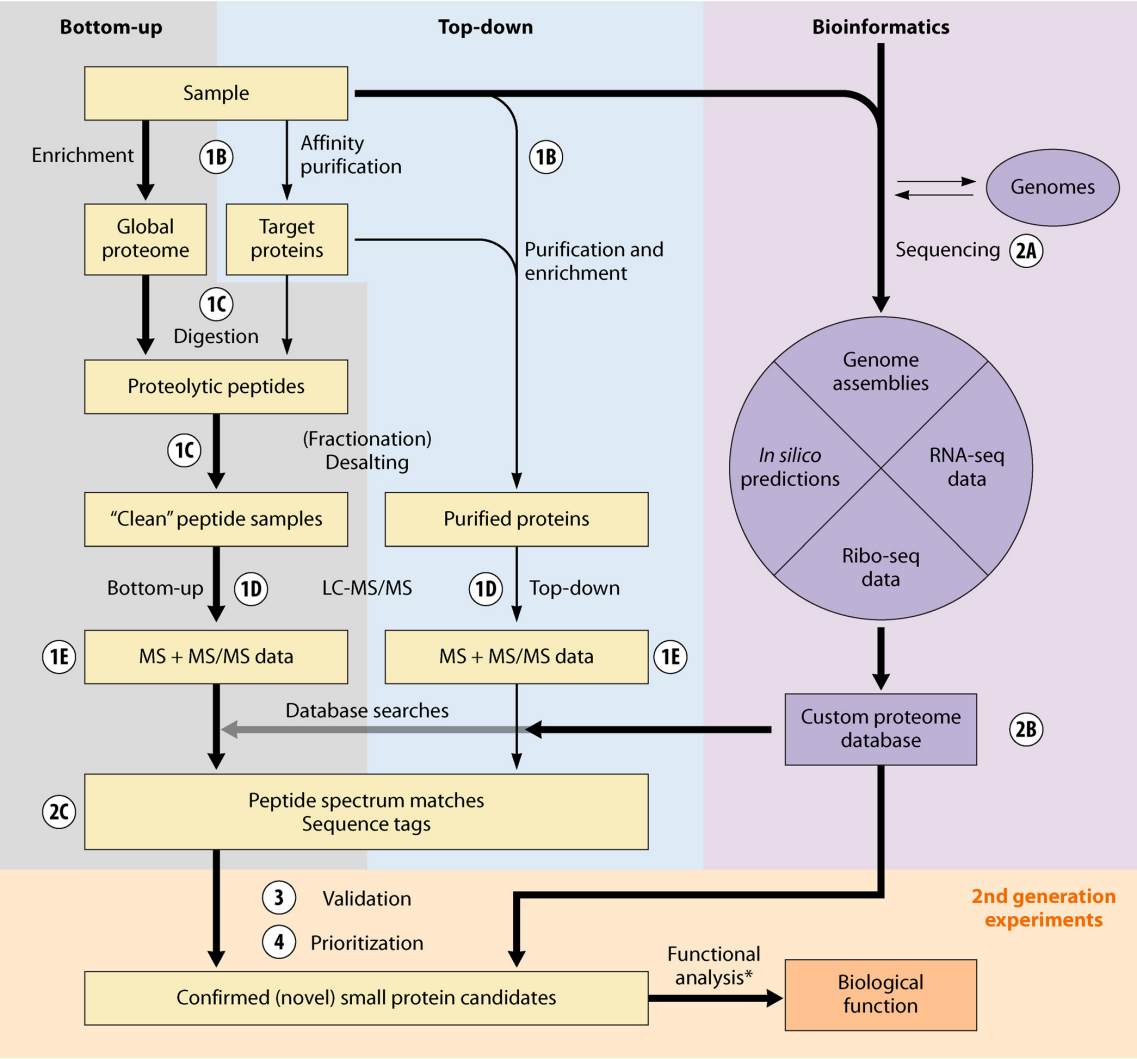
Overview of the main mass spectrometry-based workflows for small protein discovery, analysis, and characterization. The large majority of studies have relied on a shotgun proteomics discovery approach (bottom-up) to identify small proteins. Top-down approaches are slowly gaining momentum but are not yet widely accessible from core facilities. Bioinformatics is important to assemble complete genomes *de novo* (at times using genomic DNA extracted from the same sample), to integrate small protein predictions with experimental RNA-seq and Ribo-seq data to create custom databases that allow the identification of novel small proteins by MS-based proteomics. Validation and prioritization facilitate focusing on the elucidation of function(s) of the most promising novel small proteins (yellow shading; see asterisk), an aspect that is described in more detail in the accompanying article “Small Proteins; Big Questions” (124). Shading matches that in Fig. 2. Corresponding text sections are indicated by white circles, as follows: 1B, “Sample Preparation and Data Collection—Preparation and enrichment for small proteins”; 1C, “Sample Preparation and Data Collection—Protease Digestion”; 1D, “Sample Preparation and Data Collection—Liquid chromatography”; 1E, “Sample Preparation and Data Collection—Ionization and data acquisition” ; 2A, “Data Analysis—Overview: the relevance of genome sequences for proteogenomics”; 2B, “Data Analysis—Creation of custom search databases”; 2C, “Data Analysis—Stringent FDR control”; 3, “Validation of Novel Small Protein Candidates”; 4, “Prioritization/Selection of Novel Small Proteins.”

Mass spectrometry-based proteomics (MS) provides a direct method to detect and quantify small proteins on a global or targeted scale (for general proteomics reviews, see references 7 and 8). In addition, MS can provide information on the different proteoforms, i.e., different forms for a protein product derived from a single gene (9), including genetic variations, splice variants (for eukaryotes), processed forms, and different combinations of posttranslational modifications, all of which can affect protein function (for selected key MS terms, please see Box 1). Many small proteins have been detected as adventitious spectra within traditionally acquired proteomes, mainly using exploratory “shotgun” bottom-up proteomics (10). In this approach, all proteins in a sample (e.g., a cellular lysate or a purified protein complex) are digested using a protease, and subsequently subjected to liquid chromatography-coupled tandem mass spectrometry (LC-MS/MS) (Box 1). Sequences of the proteolytic peptides are inferred from their MS/MS spectra by matching the fragmentation patterns to theoretical spectra of a reference proteome database that contains the sequences of all annotated proteins of the target organism (Fig. 1). However, there are two caveats: First, MS-based detection is “amplification-free” and intrinsically limited by the sensitivity of the instrument and its dynamic range. This leads to preferential detection of more abundant proteins, and proteins that have peptides with high ionization efficiencies. Second, “conventional” bottom-up proteomics approaches are biased toward studying proteins with molecular weights above 10 kDa, which represent roughly >90% of annotated proteomes (as determined from an analysis of 21,400 complete prokaryotic genomes from NCBI’s RefSeq). Conventional studies typically report a statistically significant under-representation of experimentally identified small proteins and are thus ill-suited for their comprehensive analysis (11, 12). In fact, limitations for small protein detection exist across the entire MS workflow: experimental sample preparation and digestion, MS detection and acquisition, database search, peptide spectrum matching (PSM), and protein identification. Protease digestion is confounded by the intrinsic scarcity of necessary cleavage sites in small proteins (see “Protease digestion” below). If no MS-detectable peptides with a length of approximately 7 to 40 amino acids (aa) are generated (this range is based on MaxQuant’s lower length and upper molecular weight limit of 4,600 Da in the first-pass search [13]), a small protein will not be identifiable via bottom-up proteomics at all, unless alternative proteases are used. Furthermore, database searches to identify proteins require accurately and comprehensively annotated genomes. This is often not the case for small proteins, as gene prediction algorithms apply varying length thresholds for genes encoding proteins below 50 to 100 aa to minimize the number of spurious, false short ORFs (sORFs) (14) (see “Data Analysis” below). In addition, the traditionally applied “two peptide rule” has required more than one unique peptide for confident protein identification (15); this, however, is ill-suited for small proteins, which, due to their small size, can often only be identified by a single peptide, resulting in a higher false discovery rate (FDR) for small proteins that needs to be tightly controlled (see “Stringent FDR control” below). Moreover, quantitation based on single-peptide-hit proteins is often neither accurate nor precise (16, 17). Standard workflows for protein identification, FDR estimation, and quantification are thus less useful for small protein discovery, and have to be modified to allow their efficient and reliable detection and analysis. Targeted proteomics approaches can fill this gap and are frequently used to validate and quantify small proteins (see “Validation of Novel Small Protein Candidates” below).

BOX 1: MASS SPECTROMETRY AND PROTEOMICS CONCEPTS AND TERMS

**General approaches.**

**(i) Bottom up.** Methods in which protein samples are enzymatically or chemically cleaved into peptides prior to MS and MS/MS analysis. Typically uses liquid chromatography (LC) to separate digested peptides prior to MS and MS/MS analysis. Typically involves fragmentation in the mass spectrometer (MS/MS) to sequence peptides.

**(ii) Top down.** Methods in which protein samples are analyzed directly in the mass spectrometer without digestion. Can involve direct infusion or LC to separate proteins prior to MS and MS/MS analysis. Typically involves fragmentation in the mass spectrometer (MS/MS) to sequence and identify the proteins. Can require specialized instrumentation or custom setup of existing instruments.

**Mass spectrometry instrumentation and acquisition.**

**(i) LC-MS.** Liquid chromatography-coupled mass spectrometry and tandem mass spectrometry (LC-MS/MS).

**(ii) MS/MS.** Typical arrangement for most MS analyses, also termed “tandem mass spectrometry” Separated proteins and/or peptides are measured in the instrument and then isolated and fragmented to record tandem mass spectra. Can be used for “bottom-up” and “top-down” approaches. Typically uses electrospray ionization (ESI) to generate ions.

**(iii) MALDI/ESI.** Ionization methods for MS. Matrix-assisted laser desorption ionization (MALDI) is rarely attached to LC systems. ESI is typically coupled to LC systems and is the dominant ionization mode for proteomics.

**(iv) DDA.** Data-dependent acquisition mode. Most commonly-used approach for proteomics studies. Candidate ions are selected based on peak intensity and resolution (= data dependent), isolated, and fragmented to provide MS/MS spectra.

**(v) DIA.** Data-independent acquisition mode. A hybrid approach where LC-MS/MS data are acquired without isolating specific ions for tandem analysis. Yields complex MS/MS spectra from multiple precursors. Since all ions are fragmented for MS/MS, no information is lost in the process, and typically data quality for quantitation is higher. Requires libraries of empirical or derived data from samples to extract identification and abundance from acquired data. Existing files can be reanalyzed with new information, as no ions are discarded.

**Data analysis terms.**

**(i) PRM.** Parallel reaction monitoring. Typical targeted acquisition mode for specific detection and robust quantification. Can involve specialized instrumentation.

**(ii) *De novo*.** Direct sequencing of polypeptides from fragment spectra via comparison of mass differences to amino acid residues.

**(iii) Database search.** Peptide and protein identification from fragment spectra by comparison of spectral patterns and fragment ions to databases of theoretical spectra/fragment masses. Redundant peptides measured from this approach are called a peptide spectrum match (PSM).

Bottom-up approaches also face the so-called “protein inference issue”: protein identifications are inferred based on the detected peptides, which can be ambiguous and match to more than one protein or isoform (18). Proteins can carry different combinations of posttranslational modifications and exist in multiple proteoforms. In bottom-up proteomics, any information on posttranslational modifications or truncations is lost if the respective segment is not covered by one proteolytic peptide. In addition, there is very limited information on the combinations of these modifications. The only way to address this issue is “top-down” proteomics (19) (Fig. 1; Box 1). This digest-free approach analyzes full-length proteins and directly provides information on the different proteoforms present in a sample. The size of small proteins makes them ideally suited for a top-down proteomics approach. This is, however, a very experimental strategy with limitations in dynamic range and sequence analysis depth; only a limited number of labs conduct top-down proteomics on a routine basis. Hence, we provide guidelines and examples for top-down approaches, but more limited in scope than those for bottom-up proteomics.

Here, we describe a selection of MS-based approaches to identify and characterize small proteins in both exploratory and targeted studies. We focus on (i) approaches for sample preparation and MS data collection for small proteins, (ii) analysis of MS data sets for small protein discovery, (iii) validation of putative small proteins, and (iv) bioinformatic and MS-based prioritization of putative small proteins. While we cannot cover all approaches due to space constraints, we provide a repertoire of approaches that can be modified for a specific research question and that optimize the output for small proteins. These methods were developed by many different groups and now help to level the playing field for small proteins.

## SAMPLE PREPARATION AND DATA COLLECTION

### Overview: general considerations.

**FIG 2:**
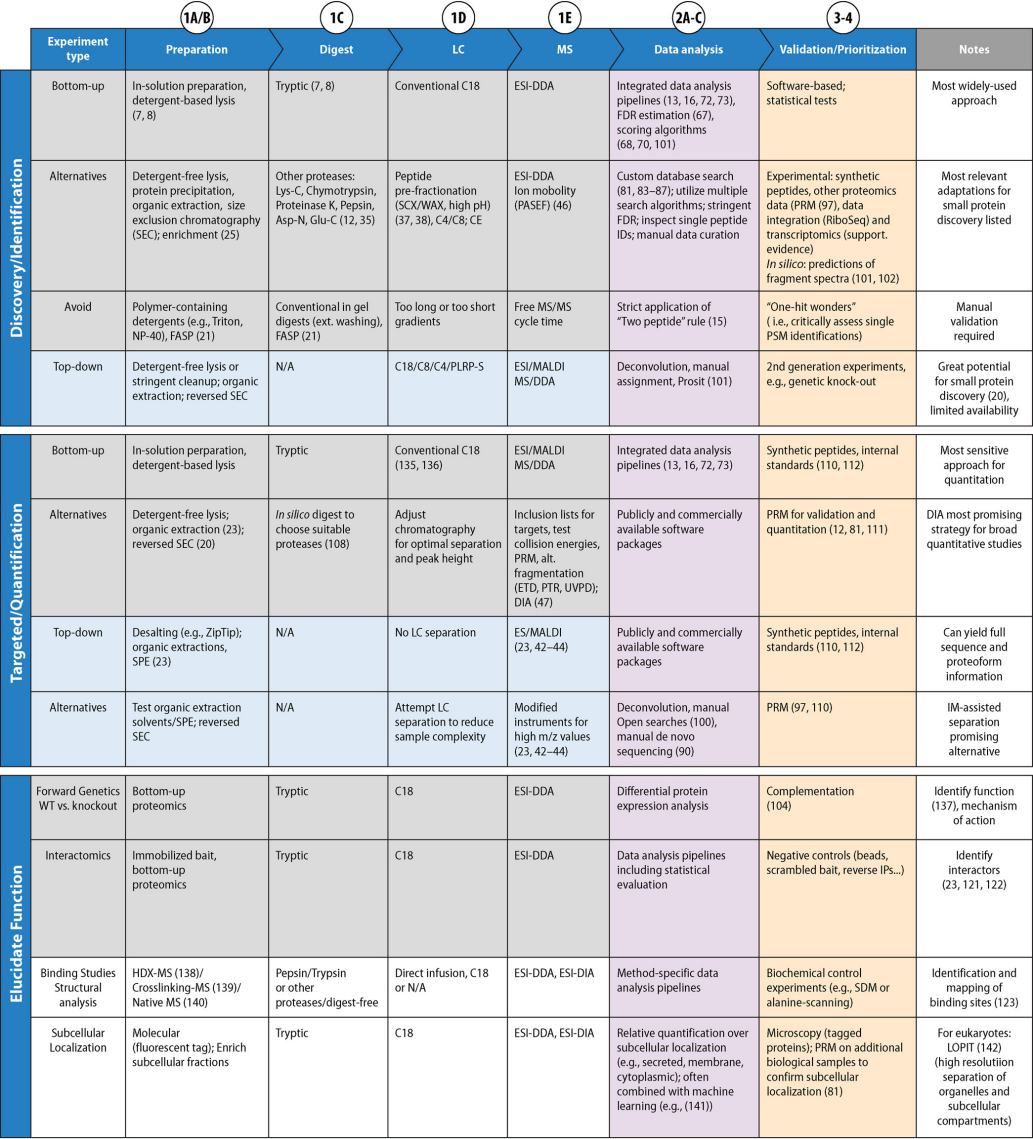
Overview of the major steps of the most common MS-based workflows for discovery/identification of small proteins, their targeted analysis (for quantification), and for the functional characterization of novel and known small proteins. The numbering of the steps is aligned with Fig. 1, with corresponding text sections indicated by white circles, as follows: 1B, “Sample Preparation and Data Collection—Preparation and enrichment for small proteins”; 1C, “Sample Preparation and Data Collection—Protease digestion”; 1D, “Sample Preparation and Data Collection—Liquid chromatography”; 1E, “Sample Preparation and Data Collection—Ionization and data acquisition”; 2A, “Data Analysis—Overview: the relevance of genome sequences for proteogenomics”; 2B, “Data Analysis—Creation of custom search databases”; 2C, “Data Analysis—Stringent FDR control”; 3, “Validation of Novel Small Protein Candidates”; 4, “Prioritization/Selection of Novel Small Proteins.” Alternative approaches are listed and selected references provided.

Small protein detection by MS is most frequently applied to discover small proteins from complex samples such as whole-cell lysates (Fig. 1; Table 1). Below, we outline the different methodological approaches for small protein discovery, highlighting differences to standard methods that are tailored to small proteins specifically (Fig. 2). We then describe parallel methods for detection of small proteins from low-complexity samples, and a separate application that robustly detects small proteins from protein complexes where a small protein has been detected, but its specific identity is unknown.

**TABLE 1:** A selection of small protein discovery studies for bacteria and archaea using shotgun (bottom-up) proteomics and top-down approaches without proteolytic digest^*a*^

Organism(s)	Taxonomy	Approach	Notes	Sample	Reference(s)
Mycoplasma pneumoniae	Bacteria	Shotgun	Term proteogenomics introduced; search six-frame translated genome	Whole cell lysate	56
Mycoplasma mobile	Bacteria	Shotgun	Used proteomics data in initial genome annotation of an organism	Whole cell lysate	126
Shewanella oneidensis	Bacteria	Shotgun	MS-based proteomics to improve genome annotation, used PTM data, studied several conditions	Whole cell lysate; subcellular fractionation	127
Methanosarcina acetivorans	Archaea	Top down	Top-down approach identified five unannotated small proteins (40–76 aa)	Whole cell lysate	19
Staphylococcus aureus	Bacteria	Shotgun	Effort to analyze the entire expressed proteome, combining different conditions and proteomics approaches	Subcellular fractionation	128
Yersinia pestis	Bacteria	Shotgun	Required 2 peptides to identify a novel protein	Subcellular fractionation	57
Mycobacterium tuberculosis complex	Bacteria	Shotgun	Custom database approach that merges information from different strains	Various	129
46 species (bacteria and archaea)	Bacteria, Archaea	Shotgun	Large proteogenomic study; use of stringent PSM level FDR advocated	Various	30
Escherichia coli	Bacteria	Shotgun	High FDR among peptides implying novel proteins; trypsin + Lys-C	Whole cell lysate	31
Helicobacter pylori	Bacteria	Shotgun	Required 2 peptides to identify a novel protein; also used size exclusion chromatography	Whole cell lysate	130
57 bacterial species	Bacteria	Shotgun	Large proteogenomics study on N-terminal methionine excision and PTM (N-terminal acetylation)	Various	131
*Saccharopolyspora erythrea*	Bacteria	Shotgun	Reannotated genome of organism with high GC content (transcriptomics, shotgun proteomics)	Whole cell lysate	58
Bradyrhizobium japonicum	Bacteria	Shotgun	Custom databases to find longer (84)/shorter proteoforms (132) (integration with dRNA-seq data)	Whole cell lysate	84, 132
*Synechococcus sp*	Archaea	Shotgun	Proteogenomic study of model cyanobacterium (8 conditions); global profiling for PTMs (1% PSM FDR)	Whole cell lysate	133
Roseobacter denitrificans	Bacteria	Shotgun	N terminomic combined with six-frame translation database to validate/correct N termini (+ alternative proteases; Glu-C, chymotrypsin)	Whole cell lysate	27
Mycoplasma pneumoniae	Bacteria	Shotgun	Integrated anaylsis to re-annotate genome (>300 transcriptome, >70 proteome datasets); evidence for internal starts, new small proteins	Various	103
Pseudomonas stutzeri	Bacteria	Top down (MALDI)	Small transmembrane subunit of cbb3 oxidase	Purified protein complex	23
Metagenomic study of grassland soil	Bacteria, archaea	Shotgun	Metagenome-assembled genomes as basis for meta-proteomics (custom database); integrate metabolomics; beyond culturable strains	Soil extract	61
Listeria monocytogenes	Bacteria	Shotgun	N-terminal enrichment (COFRADIC approach) (26); 2nd study (92) used spectral libraries and combined DDA and DIA	Whole cell lysate	26, 29, 92
Xanthomonas euvesicatoria	Bacteria	Shotgun	Reannotation of a plant pathogen with Shotgun data; confirmed expression of 5 novel proteins with Immunoblot (c-Myc tag)	Whole cell lysate	134
Bartonella henselae	Bacteria	Shotgun	Broadly applicable proteogenomic approach, custom databases validated 107/138 peptides with PRM (97)	Subcellular fractionation	81, 97, 141
Methylobacterium extorquens	Bacteria	Shotgun	N terminome study of a strain used for microbial rehabilitation and degradation of industrial pollutants	Whole cell lysate	28
Escherichia coli	Bacteria	Top down (MALDI)	Small transmembrane subunit of bd oxidase	Purified protein complex	43
Bacillus subtilis	Bacteria	Shotgun	Explored small protein enrichment strategies, different proteases, database searches; validation by PRM and spectral matching	Small protein enrichment	12
Salmonella Typhimurium, Deinococcus radiodurans	Bacteria	Shotgun	Broadly applicable custom peptide DB; integrated Ribo-seq data and peptide fragmentation prediction	Whole cell lysates	88
Salmonella Typhimurium	Bacteria	Shotgun	Integrated small protein prediction with Ribo-seq, shotgun, and other OMICS data; elucidated function of novel small proteins	Various	104
Prokaryotes from human microbiota, Bacteroides thetaiotaomicron	Bacteria, archaea	Shotgun	Prediction of ∼4500 small protein families <50 aa; experimental evidence for selected examples (meta-transcriptomics/proteomics) Identified several small proteins in Bacteroides thetaiotaomicron	Various	1
Intestinal microbiota model system	Bacteria	Shotgun	Extended custom iPtgxDB to multispecies model (8 strains); meta-transcriptomics + meta-proteomics; spectral matching	Small protein enrichment	60
Thermosynechococcus elongatus	Bacteria	Top down (MALDI)	Small transmembrane subunit of photosystem II	Purified protein complex	44
Staphylococcus aureus	Bacteria	Shotgun	Broadly applicable approach; integrated shotgun and Ribo-seq data; automated evaluation of MS/MS spectra quality	Cytoplasmic extract	87
Methanosarcina mazei	Archaea	Shotgun/top down	Characterization of 36 proteoforms mapping to 12 small proteins with top down (2D-LC-MS)	Small protein enrichment	89
Methanosarcina mazei	Archaea	Shotgun	Multiprotease approach (SDS-PAGE)	Small protein enrichment	35

a We apologize to authors of the many important studies we could not reference due to space restrictions. Preference was given to more recent studies, many of which have used higher accuracy MS instruments and small protein enrichment strategies, carried out validation of novel small proteins (e.g., by PRM or spectral matching), and put a larger emphasis on integration of RNA-seq, Ribo-seq or computational small protein prediction algorithms. Proteogenomic studies prior to 2014 are more thoroughly listed by Kucharova and Wiker (125).

An important consideration in method choice is the use of bottom-up or top-down proteomics (Box 1). The vast majority of proteomics studies make use of bottom-up methods, although top-down approaches are ideally suited for small proteins. However, practical considerations in method choice are often limited by the expertise and instrumentation available in a core facility. Bottom-up methods are widely available at high quality in most laboratories and core facilities, and sample introduction from bottom-up small protein preparations can be reasonably approached by routine-use setups. In general, we recommend bottom-up analyses for complex samples and top-down approaches for low-complexity samples, due to the ease of data acquisition of the two approaches. Low-complexity samples can also be analyzed directly using matrix-assisted laser desorption ionization–time of flight (MALDI-TOF) mass spectrometry, providing direct information on the different small proteins present in a sample and their molecular weights.

Below, we outline general considerations for each step of an MS-based analysis of a sample for small proteins. The experimental workflow can be divided up in four parts (Fig. 2), described in turn below.

### Preparation and enrichment for small proteins.

Depending on sample complexity and the type of MS analysis, different preparation steps are required. In general, bottom-up analyses focus on efficient denaturation and digestion, while top-down approaches mainly utilize size exclusion steps to enrich small proteins (20). We outline the advantages and limitations below, and suggest modifications to existing protocols as well as alternative strategies (Fig. 2).

**(i) Sample preparation for bottom-up proteomics.** Sample preparation for bottom-up proteomics typically includes denaturation, reduction, and alkylation steps to ensure optimal access of the proteases to unfolded proteins, and to prevent thiol oxidation and disulfide bridge formation. *Per se*, these steps do not impede small protein detection; however, most protocols were designed for protein molecular weights >10 kDa, and include efficient removal steps for small molecular weight contaminants. In particular, filter-based methods such as filter-aided sample prep (FASP) (21) and suspension traps (S-Traps) (22) efficiently remove detergents and contaminants prior to digestion, leading to improved data quality and signal-to-noise ratios for studies of large proteins and proteomes. However, these approaches, if used conventionally, also actively de-enrich small proteins (Fig. 2). We therefore suggest using either in-solution digests without any cut-off filters, or modifying solid-phase-assisted digest protocols to avoid removal of small proteins by reducing stringency in the wash steps, described below in “Protease digestion.” However, if sample composition requires extensive washing, small protein detection remains challenging using this approach.

**(ii) Sample preparation strategies using protein precipitation steps.** The same considerations apply for sample preparation strategies using protein precipitation steps that are often applied to concentrate and purify target proteins, e.g., organic solvent precipitation using acetone, methanol, chloroform or other solvents (Fig. 2). Small proteins may remain in the soluble fraction due to their high solubility in organic solvents, and hence may be discarded. We recommend avoiding precipitation steps to ensure small proteins are not lost. Despite the challenges associated with organic extraction, membrane proteins benefit from organic extraction due to their high hydrophobicity. Moreover, top-down approaches benefit from organic phase extractions, as detrimental salt adduct formation is minimized. Our and other labs found that a combination of low organic solvent concentration with organic solvent-resistant filter membranes identified peptides that were not accessible using conventional bottom-up analysis, thereby significantly expanding the identified small proteome in different organisms (23, 24).

**(iii) Enrichment using typically discarded fractions.** While the above modifications reduce the likelihood of de-enriching small proteins, it is also possible to actively enrich small proteins by using only the fractions that are typically discarded in a conventional bottom-up proteomics preparation. This can be achieved by either (i) using molecular weight cut-off membranes, with large proteins being trapped using a filter-membrane with a low molecular weight cutoff, and the flow-through containing small proteins being retained for subsequent analysis, or (ii) subjecting the sample to a standard organic precipitation; the precipitated proteome is discarded, and the supernatant is digested and submitted to LC-MS/MS analysis. Both of these techniques can also be applied to top-down approaches. The small protein fractions isolated from these approaches typically coenrich for other interfering small molecules from the sample and common preparative contaminants. Hence, special care must be taken to remove these prior to MS analysis. In addition to reverse-phase desalting prior to analysis, we recommend desalting using strong cation exchange by solid-phase extraction (SCX-SPE).

**(iv) MS approaches.** MS approaches commonly involve a prefractionation step to reduce sample complexity, with the most widely used methods being separation of the sample by (i) centrifugation, into soluble and membrane fractions; (ii) sucrose density gradients, based on hydrodynamic radius; (iii) size exclusion or ion-exchange chromatography; and (iv) SDS-PAGE. These methods can be applied to study small proteins, but separation by SDS-PAGE requires modification to prevent loss of small proteins from the sample; small proteins often stain poorly with Coomassie, or pass through the gel with minimal retention. Isolating the dye front (typically bromophenol blue) and a narrow range of low MWs will enrich small proteins. If no bands are detected using Coomassie staining, we suggest silver staining due to its higher sensitivity. After excision of the respective gel areas, we recommend only minimal wash steps, as small proteins may diffuse out of the gel into the wash buffer. When small proteins are not efficiently recovered with these conventional gel-based approaches, distinct MW regions can also be selected by electroeluting specific regions or by gel-eluted liquid fraction entrapment electrophoresis (GELFrEE) and downstream processing in the liquid phase (25).

Another useful biochemical purification strategy is a combined fractional diagonal chromatography (COFRADIC) approach (26), where chemical or enzymatic modification prior to and/or after the digestion step allows for specific enrichment for a certain peptide fraction, e.g., N-terminal peptides (Table 1), which greatly reduces the sample complexity. This method requires a suitable database containing alternative start sites (see “Creation of custom search databases” below) but then can provide direct information on translational start sites (27–29).

In general, we recommend fewer preparation and manipulation steps for less complex sample mixtures prior to MS analysis. For purified protein complexes, we suggest minimal desalting and purification using, for example, reversed-phase tips or cartridges, with samples then analyzed directly by MALDI-MS or -MS/MS. For complex protein mixtures and lysates, we recommend modifying S-trap protocols with minimal washing, organic extractions, or reciprocal purification using molecular weight cut-off filters.

### Protease digestion.

Protease digestion is the defining step of bottom-up proteomics. Digestion is often performed in-solution, in-gel, or during filter-based sample preparations. These digestion protocols use extensive washing of the sample that inherently de-enriches small proteins; due to their size, small proteins can pass through filter membranes, may not adsorb sufficiently (or may stick too tightly) to solid phases, and may be flushed during wash steps. Hence, the digest should be conducted in a way that specifically ensures inclusion of, and access to, small proteins, which can range from in-gel and in-solution digests to solid-phase supported protocols. The advantages and limitations of different digestion strategies for small proteins are detailed below.

Digestion of small proteins is challenging due to the intrinsic scarcity of necessary protease cleavage sites, and the often short length of the few peptides that are generated. In general, Trypsin, mostly alone or in combination with LysC, has been the workhorse for bottom-up proteomics due to cleavage specificity (cleaves after K and R) and activity in typical preparation conditions. This excellent enzyme/combination of enzymes, in particular in consecutive denaturing conditions, has also been widely used to identify small proteins in different prokaryotes (30, 31) (Table 1). Alternatively, proteases targeting other amino acids, such as Asp-N and Glu-C, are used for studying posttranslational modifications such as K acetylation (32), or for specific target protein groups (33). However, the high specificity of these proteases limits their activity against small proteins, and specificity for basic amino acids limits activity against small membrane proteins in particular, which are deficient in charged residues in membrane-spanning regions. Proteases with specificity for other amino acids, or lower specificity, such as chymotrypsin (mainly F, Y, and W > M and L), proteinase K (hydrophobic amino acids), pepsin (nonspecific at pH <2), elastase (A, V, S, G, L, and I), or subtilisin A (nonspecific) (34), are promising alternatives for identifying small membrane proteins (35) (Fig. 2). However, using a protease with lower specificity comes with several challenges: (i) proteases with limited specificity require careful testing and optimization of protease:protein ratio, digestion time, digestion buffer, and digestion temperature; (ii) the abundance of each proteolytic peptide species will be lower due to the statistical nature of the cleavage, i.e., each protein is potentially cleaved into multiple, different, overlapping peptides, each lower in abundance compared to a specific digest where only a single peptide is produced from each segment; (iii) the lack of a charged amino acid at the C terminus will lead to MS/MS spectra with a lower number of usable fragment ions; (iv) the search space for database matching is increased substantially, with consequences for the FDR of protein identification (see protease: protein “Stringent FDR control” below); and (v) nonspecific cleavage compromises stoichiometric recovery, limiting quantitation of peptides and proteins. We therefore recommend to initially use trypsin/LysC as proteases for bottom-up experiments, except if membrane proteins are targeted specifically. The choice of enzyme largely depends on the sample and the small proteins of interest: some hydrophobic small proteins still yield proteolytic peptides with high-quality, information-rich fragment spectra (36), while other small membrane proteins are completely inaccessible to bottom-up approaches and require top-down analysis (23). If trypsin/LysC proves ineffective, in particular for samples with low complexity, alternative proteases can be tried, as described above (12, 27, 35). After successful digestion, regardless of the protease used, proteolytic peptides in very complex mixtures can be further fractionated prior to LC separation for more sequencing and data depth (37, 38).

### Liquid chromatography.

In bottom-up methods, proteolytic peptides are typically separated by liquid chromatography prior to acquisition of MS (ionized peptide precursors) and MS/MS data (fragmentation pattern of peptides) (135, 136). The most widely used solid phases for separation are different derivatives of C_18_ bed materials (Fig. 2) that separate the peptides based on hydrophobic interactions with the column bed, and a gradient elution of aqueous into hydrophobic solvent. A typical LC-MS setup might include a trap column to facilitate removal of small molecular weight contaminants prior to separation on the main chromatographic column. Current LC-MS setups for bottom-up proteomics are optimized for peptide lengths between 8 and 25 aa. They will thus perform equally well for proteolytic peptides as well as for short small proteins, and will also work well for top-down experiments for many small proteins. Depending on their amino acid composition, however, small proteins >40 aa may not elute efficiently from a conventional C_18_ column, even at high concentrations of organic solvent. Membrane proteins also often bind strongly to C_18_ phases due to their high hydrophobicity, and even small membrane protein-derived peptides may stick to the C_18_ phase irreversibly. In these cases, other bed materials with lower hydrophobicity (C_4_/C_8_) and larger pore sizes (>300 Å) may offer higher recovery in bottom-up and top-down experiments. This often comes at the cost of decreased retention and recovery of hydrophilic peptides and small proteins.

For the analysis of complex bottom-up or top-down samples, extending the length of the chromatographic gradient may offer significant improvements in data depth, as more acquisition time is available (39). However, gradient length should be adjusted to fit the complexity of the sample: chromatographic peaks broaden with increasing overall gradient length, and the associated decrease in intensity per spectrum impairs detection and analysis of low-intensity candidate ions. In general, we recommend short gradients (15 to 60 min) for samples with low complexity and long gradients for samples with high complexity (90 to 180 min). The chromatographic separation of peptides and small proteins may well be further improved in the future by different bed materials, narrow column diameters, or micropillar array columns (40, 41).

### Ionization and data acquisition.

Most proteomics labs operate electrospray ionization (ESI)-based mass spectrometers due to their direct interface with LC and high ionization efficiency. This combination allows fast acquisition of high quality data, and is equally applicable to conventional bottom-up proteomics approaches and top-down experiments (Fig. 2). ESI-based ionization only falls off for membrane proteins that are highly hydrophobic. For dedicated small membrane protein studies, MALDI ionization can be a promising alternative, although MALDI data acquisition to LC is time and cost intensive and rarely used.

For low-complexity samples, we recommend performing MALDI-MS measurements to get an initial overview of small proteins present. In MALDI-MS, samples are spotted in a solid matrix on a conductive plate and ionization is achieved using a laser that transfers energy via the matrix molecules to the proteins of interest. MALDI usually imparts lower charge states on the analytes and thus makes MS and MS/MS spectra much easier to interpret than ESI spectra, where most peptides typically carry 3 to 5 charges. MALDI spectra can also provide initial information not only on the molecular weight of any candidate small proteins, but also whether multiple candidates are present that cannot be separated efficiently using SDS-PAGE. Sample preparation could either use organic extraction or a desalting step using either C_18_ (soluble small protein) or C_4_/C_8_ (membrane small protein) tips or SCX-SPE-based desalting as outlined above. After solvent removal, these purified complexes can then be directly spotted and submitted to MALDI-MS analysis. If a MALDI-MS/MS instrument with sufficient isolation power and resolution is available, small proteins can also be identified directly from these spectra (23, 42–44). If protein identities cannot be directly determined, MALDI-MS survey spectra can already yield valuable information on the number of small proteins and their proteoforms in a sample.

For LC-MS/MS analysis of low-complexity samples, more measurement time in an LC-MS experiment can be spent on each candidate ion. We therefore recommend testing multiple collision energies and other “slow” fragmentation methods (ETD/ETHCD/UVPD/PTR) to generate complementary data, and increase the fragment coverage of the small proteins of interest.

We anticipate that ion mobility-coupled separation of ions will improve the scope and depth of conventional Data Dependent Analysis (DDA)-based bottom-up proteomics analyses also for small proteins (45, 46). In addition, data-independent analysis (DIA) based methods may well further improve bottom-up and top-down analyses of small proteins in the future (47) (Box 1). DIA is an acquisition approach where large *m/z* ranges are sequentially fragmented independent of precursor abundance, providing information-rich MS and MS/MS data sets that can be mined. Since no precursors are “discarded,” presumably numerous undiscovered small proteins reside in the data awaiting detection. This approach, facilitated in recent years by faster and more sensitive instruments, is expected to benefit substantially from further improvements in analytical pipelines.

## DATA ANALYSIS

### Overview: the relevance of genome sequences for proteogenomics.

Unbiased identification of small proteins from MS data requires a reference genome sequence. Owing to advances in DNA sequencing technologies and assembly algorithms, complete genome sequences can be readily and cost efficiently assembled *de novo* (48, 49). Complete genomes provide an optimal basis for functional genomics (50) and for small protein discovery. They can be used to create both reference or custom search databases that link to genomic coordinates (e.g., NCBI’s RefSeq or Ensembl), an advantage over the widely used universal protein (UniProt) database that provides additional functional information and annotations but lacks the link to the genome sequence (51). Notably, advances in genome annotation have lagged behind the sequencing revolution (52), and several unresolved issues remain. These include discrepancies in the number of protein coding sequences (CDSs) predicted by different reference annotation centers for the same genome sequence, discrepancies with respect to the precise protein start sites (53, 54), and underrepresentation of genes encoding stably expressed and functional small proteins, i.e., false negatives (55). Furthermore, prediction of some spurious ORFs (false positives) (11, 56, 57) is another issue, especially for genomes with a high GC content, e.g., many actinomycetes (58). As current gene prediction algorithms cannot separate truly coding sORFs from the overwhelming majority of spurious, random sORFs (14), varying size thresholds between 50 and 100 amino acids have been used to minimize the number of false small protein predictions in genome annotations. As a standard database search of MS data will only identify proteins contained in the list of annotated genes, custom databases are needed for small protein discovery (Fig. 1).

Initiatives like the Genomic Encyclopedia of Bacteria and Archaea will significantly boost small protein studies in taxonomically diverse organisms (59). We expect more studies to explore novel small proteins from moderately complex samples such as synthetic consortia (60) and from complex metagenomes (1, 61) (Table 1), based on as-complete-as-possible metagenome-assembled genomes (62, 63).

Proteogenomics, a term first coined in 2004 (56), refers to the use of MS data to provide expression evidence for CDSs missed in genome sequences (for reviews, see references [Bibr B64][Bibr B65][Bibr B66]). Searching tandem MS/MS data against one of several different flavors of custom databases allows identification of small proteins plus novel start sites and evidence for expressed pseudogenes, which would be missed if using standard reference databases; for a concise selection of small protein discovery studies using this approach in bacteria and archaea, see Table 1. Several data analysis solutions have been developed that represented important advances for the proteomics field in general and improved overall data quality: (i) search strategies that allow estimation of the number of false positive PSMs and the FDR, including so-called “target-decoy searches,” where a peptide spectrum that matches a decoy sequence such as a reversed or randomized protein sequence is considered a false positive identification (67); (ii) statistical models that employ different scoring functions or machine learning approaches to improve the accuracy and robustness of the automated PSM step, i.e., that maximize the number of confident peptide matches with minimal false positive rates (68–70); and (iii) protein inference algorithms that deduce which proteins (or protein groups) were initially present in a sample based on the experimentally observed peptides, many of which (especially in eukaryotes, but also in custom databases [see below]) are ambiguous and imply several proteins (71). Integration of these tools into publicly available data analysis pipelines for shotgun MS data has enabled researchers to carry out large parts of the data analysis themselves (13, 16, 72, 73) (Fig. 2). However, even when using highly accurate MS instruments and optimized small protein sample preparation strategies (12, 25), MS-based novel small protein discovery is still challenging and requires custom databases and a stringent control of the FDR.

### Creation of custom search databases.

All customized databases add short ORFs that potentially encode true small proteins. Most often, custom databases are based on a six-frame translation of the genome sequence. While this approach guarantees inclusion of all possible gene products, it creates a substantially larger search space compared to the standard reference search database: typically, 20 to 50 times more proteins and ∼4 to 8 times more distinct tryptic peptides (74). Hence, it requires substantial analysis time to identify the respective reading frame and the precise novel small protein boundaries, and to ensure that the peptides are unique (i.e., unambiguously identify one protein). Consequently, various approaches have been developed that aim to reduce the search space. To limit the database size and thereby improve PSM statistics (75), transcriptomic or Ribo-seq data from the conditions of interest can be used to create a smaller database that only considers the genomic regions transcribed or translated. Tailored transcriptome- or Ribo-seq based databases were successfully used to identify small proteins in different eukaryotes (76–79) and prokaryotes (80). For more complex eukaryotes, where larger numbers of protein families and splicing lead to a substantial percentage of peptides being ambiguous, this is a promising approach that simplifies protein inference. However, for prokaryotes that lack splicing and have smaller genomes, creating a single database applicable to all conditions is more versatile, and allows identification of small proteins that are conditionally expressed and hence could be missing from RNA-seq or Ribo-seq data. We recently developed an approach to address the dilemma of largely differing genome annotations, missing sORFs, and the higher fraction of ambiguous peptides in large custom databases, by constructing “integrated proteogenomics databases for the protease of choice” (iPtgxDBs) (81). In a pre-processing step, all annotation sources (reference annotations, *ab initio* gene predictions, and a modified form of a six-frame translation that considers the most common alternative start codons GTG, TTG, and CTG [82]), plus all proteins down to a user-specified length threshold, are integrated and consolidated, capturing both overlap and differences. By adding peptides that imply potential new start sites, an iPtgxDB can be kept minimally redundant; it contains ∼1/3 fewer peptides than a 6-frame translation. Notably, up to 95% of the peptides unambiguously match one protein. For more information about preprocessing and a public web server to create such iPtgxDBs, see https://iptgxdb.expasy.org. Several other public proteogenomics software solutions or custom search database approaches have been developed to support researchers with small protein identification (83–87), some of which also provide the useful option for integrative data visualization in a genome viewer, or include an option to consider predicted peptide ion intensities (88). The more recent proteogenomics studies emphasize efforts for data validation and integration of orthogonal data sets (see Table 1 for some examples). Top-down studies where novel small proteins were identified via a custom database search are slowly gaining momentum (Table 1) (19, 23, 89). In addition, one can also rely on top-down data in combination with *de novo* sequencing, a completely database-independent approach that infers small protein sequences by searching for amino acid-specific mass increments between adjacent fragment peaks (90). We expect that improvements in the accurate and comprehensive prediction of small proteins from DNA sequence alone will facilitate the creation of more focused databases, thereby also improving the PSM statistics.

### Stringent FDR control.

A second key consideration for small protein discovery concerns false discovery rate (FDR) control (Fig. 1). Historically, more confident protein identification from MS/MS data has relied upon the “two-peptide rule” (see the introduction and reference 15). However, this rule is ill suited for small proteins, which, due to their small size, are often only identified by a single peptide (91, 92). To minimize the identification of so-called “one-hit wonders,” several PSMs (independent observations of the same peptide sequence) should be required. As small proteins are often of lower abundance (81, 93) and hence are expected to produce few MS-detectable peptides, researchers have analyzed multiple biological conditions to increase their odds of identification, and used proteases other than trypsin (Table 1) that can add more spectral evidence to support novel small protein identification (12, 35, 60). It is important to note that the number of false positive identifications will also increase as the size of the MS data set increases (94, 95). We suggest performing the steps outlined below before assessing any potential novel hits, as it can be rather discouraging to see your “top novel small protein candidates” fail to withstand rigorous evaluation.

Another critical consideration when interpreting FDR estimates from MS data analysis is that the false positive protein identifications are distributed unevenly between large (mostly annotated) and small (mostly unannotated) proteins; custom databases will contain many more predicted small proteins than annotated proteins, such that the likelihood of a PSM matching a predicted small protein by chance is much higher than the likelihood of matching an annotated protein by chance. Moreover, there will be proportionally more PSM matches to annotated proteins than to small proteins, causing the overall FDR estimate to be skewed toward the value for annotated proteins. Consequently, reported protein-level FDRs substantially underestimate the true FDR for novel small proteins (71, 74, 94, 95). We and others have advised to strictly control the PSM FDR to 0.1% or (for very large data sets) even lower (30, 81), in order to achieve an estimated protein-level FDR for small proteins of around 1% (Table 1). Alternatively, if a more relaxed FDR is applied, the potential for false positives can be addressed with more extensive validation experiments (see below).

We also recommend applying a resource-dependent filter on top of the global FDR filtering step, as proposed earlier (64), which essentially requires more spectral evidence from less credible prediction sources for novel small proteins, e.g., from *ab initio* gene predictions and a six-frame translation (74); we have used thresholds of 2 PSMs for peptides implying RefSeq-predicted proteins, 3 PSMs for peptides of candidates from the excellent *ab initio* gene predictor Prodigal (96), and 4 PSMs for *in silico* ORFs solely predicted based on potential start codons. These values were in part motivated by a study on Bartonella henselae (81), where we successfully validated a novel small protein identified by one peptide and 3 PSMs in a very large data set with parallel reaction monitoring (Box 1) (97). Moreover, we recommend considering aspects like repeated identification in biological replicates, high spectral quality score (e.g., using the MS-GF+ search engine scores [98]), *q* values (a statistical confidence measure provided along with the posterior error probability by Percolator [99]), and second peptide searches, which can identify less abundant peptides in chimeric spectra (overlapping fragmentation patterns of co-eluting peptides), potentially including novel small proteins (88). As proteomics workflows rely on database searches, in some cases, modified peptides of abundant proteins may represent a better match than the top-scoring PSM implying a novel small protein. They were not considered because the modification was not specified in the database search; typically, only few modifications are specified in closed searches to keep the search time manageable. Fast open search software solutions like MSFragger (100) represent a valuable option to identify and eliminate such false positives.

Recently, software tools were developed that accurately predict the retention time of peptides and peptide fragmentation intensity purely *in silico* with very encouraging results for large proteomics studies and improving the number of correct PSMs (101, 102). In the near future, we foresee that such tools will also allow users to increasingly leverage data from data independent analysis (DIA) workflows.

## VALIDATION OF NOVEL SMALL PROTEIN CANDIDATES

### Overview: options for validation.

Newly identified small protein candidates from MS or non-MS data, such as RNA-seq, Ribo-seq, computational prediction, or genetic inference, need to be validated before they can subsequently be prioritized for further study (Fig. 1). As genome/proteome-scale methods identify false positives as described above, it is critical to provide independent lines of evidence to support the existence of a novel small protein identified by these methods. Transcriptomic data (ideally obtained from the same samples used for proteomic analysis [11, 81, 103, 104]) can be considered supporting evidence for MS-identified small proteins, with products of strongly expressed genes more likely to be detected at the protein level; however, not all RNAs are translated into a stable or MS-detectable protein. (105). Ribo-seq data can also complement MS-based evidence (104), and are particularly well-suited for identification of protein start sites (6, 93, 106), which is more challenging with MS-based approaches. Fifteen out of 16 novel small proteins jointly implied by Ribo-seq data and predicted by sPepFinder (107) in Salmonella enterica serovar Typhimurium were validated by MS, highlighting the complementarity of MS and Ribo-seq as methods to identify small proteins (104). Even more valuable than Ribo-seq data is direct evidence for the expression of a small protein, which can be obtained using classical immunochemistry such as Western blot analysis. However, either antibodies would have to be raised against each candidate, or a chromosomal tagging approach that introduces small sequence tags would need to be carried out (106). A simpler alternative is to use additional MS-based approaches that can be applied on a larger scale.

### MS-based validation of small proteins identified using non-MS methods.

For MS validation of putative small proteins identified by non-MS methods, we recommend using the approaches described above for sample preparation and MS data collection. Since the sequence of the putative novel small protein is known, protease cleavage prediction tools such as “Peptidecutter” (UniProt) or “ProteaseGuru” (108) serve as valuable tools to choose a suitable protease that produces detectable proteolytic peptides. Additionally, *in silico* prediction of the proteolytic peptides, based on their sequence, can be used to reanalyze previously acquired data to look for any signals in the respective retention time and *m/z* windows as a primary indication if the candidate peptides were present in the first place (101, 109). However, to date, *in silico* predictions only work well for tryptic peptides (101), so this approach cannot be used reliably for targets requiring alternative proteases or for top-down approaches. If a signal is detected, targeted methods like parallel reaction monitoring can be used to selectively accumulate and fragment ions in the specific retention time and *m/z* windows. In parallel reaction monitoring, specific RT and *m/z* values are generated for synthetic peptides that uniquely identify novel small proteins (110) to ensure that the recorded transitions do not stem from a coeluting peptide from another protein (111).

### Validation of MS-identified small proteins using synthetic peptide mimics.

For small proteins identified from complex samples by MS, a broadly applicable and economic approach for validation is to purchase synthetic peptides that match the putative small protein(s), determine their retention time, capture their fragmentation patterns, and compare these spectra to the experimentally observed small protein spectra. High-scoring matches add confidence to the initial MS identifications (60). Due to the relatively low cost of peptide synthesis, this approach can be applied to confirm a large number of proteins, thereby supporting the option of using a less stringent FDR for initial data analysis, and investing more effort into the validation. In a large study on B. henselae, overall almost 80% of 136 peptides implying novel sORFs, novel start sites, or expressed pseudogenes, were successfully validated by parallel reaction monitoring, with lower success rates for N-terminal extensions and *in silico* ORFs (81). Alternatively, databases of spectra associated with specific peptides (spectral libraries) can be generated from experimental data (typically DDA data, which results in cleaner, higher quality reference spectra) and used to more robustly identify and quantify candidates with low signal intensities or incomplete fragment spectra. However, in a study of Bacillus subtilis, parallel reaction monitoring-based validation proved more discriminatory than a spectral library approach (12).

A modification of this approach that is more sensitive but also more expensive, is based on synthetic, “heavy” isotope-labeled “AQUA” peptides (112). These heavy peptides can be used both for validating small protein candidates as well as for quantitation of small protein candidates with low signal intensities or insufficient fragment spectrum data for an unambiguous identification, but have to be designed and synthesized for each small protein of interest. The heavy peptides are added to samples containing the putative small protein(s) of interest, and provide a direct reference for retention time, signal intensity, and fragmentation pattern, facilitating unambiguous verification of novel small proteins.

## PRIORITIZATION/SELECTION OF NOVEL SMALL PROTEINS

There is increasing evidence that some, perhaps many, small proteins that can be detected by transcriptomic or MS-based approaches are nonfunctional, as defined by the lack of an effect on cell fitness (6, 93, 113, 114). Hence, prioritization of novel small proteins for further study is a critical step (Fig. 1). Classical annotation approaches that use similarity to functionally characterized proteins are generally ineffective for small proteins due to their size. For example, the overall percentage of CDSs annotated as hypothetical (i.e., lacking any functional annotation) is around 12% (based on a meta-analysis of ∼21,400 completely sequenced prokaryotes and 80 million encoded proteins). For the subsets of CDSs with a length below 100 aa or below 50 aa, this percentage rises to 41% for each subset, respectively. Similarly, in a landmark study, 4,500 conserved small protein families were identified using comparative genomics of metagenomic data sets, but 90% of the families lack a predicted protein domain, and 50% were not even annotated in reference databases (1). Important caveats are that the ability to predict a domain is somewhat dependent on protein size, and the lack of a predicted protein domain does not necessarily indicate the lack of a biological function. Small protein functional annotation is expected to improve as more small proteins are characterized, but alternative approaches for prioritization are recommended.

As an initial prioritization step, we recommend searching databases of protein annotations (e.g., eggNOG, whose predictions can be retrieved for a particular taxonomic level [115]), domains/motifs (e.g., InterProScan or LipoP [116, 117]), and predicted protein-protein interactions (118). The large majority of small proteins are not expected to generate significant hits with these tools due to the small protein size, but this is an extremely easy approach to try, and it has proven to be effective in a few cases. For example, two novel small proteins identified in B. henselae were predicted by LipoP (117) to contain signal peptides for the Sec/SRP secretion machinery, and both proteins were experimentally detected in membrane fractions and later validated with parallel reaction monitoring (81).

Analysis of phylogenetic sequence conservation, including determining the ratio of synonymous to nonsynonymous SNPs between homologues, is highly recommended for prioritization of novel small proteins, since evidence of purifying selection supports a functional role (1, 93, 113). The genomic context of the candidate ORFs is yet another important consideration, since short ORFs overlapping larger genes may be conserved due to selective pressure on the overlapping gene. Genomic context can also be informative for predicting the specific functions of small proteins or short ORFs, although providing evidence for function *per se* (rather than a specific function) is an important prerequisite. Short ORFs immediately upstream of genes/operons may function to regulate downstream transcription or translation (119). Short ORFs can also encode proteins that have related functions to the proteins encoded by the neighboring genes, such as a novel ribosome-related small protein family identified in the human microbiome study, where the novel small protein was encoded just downstream of two annotated ribosomal proteins (1). We therefore recommend using model organism resources and databases that contain operon assignments and predictions.

An additional level of prioritization can be achieved by integrating differential gene and protein expression data, where available (104). Regulated expression provides evidence for function, but can also suggest specific functions, based on the conditions and strains tested. Lastly, codon usage can provide evidence for function, since codon sequences are non-random with respect to the nucleotide content of the genome (93, 120). This is particularly true for genomes with extreme A/T or G/C content. As for analysis of sequence conservation, care must be taken to analyze only the codon usage of ORF regions that do not overlap other genes.

Each of the approaches described above can provide evidence for small protein functions that can be used for prioritization. However, we recommend relying on multiple, independent lines of evidence, since any individual method is likely to generate a substantial number of false positives. It is also the case that the statistical power of analyses of sequence conservation and codon usage is strongly length-dependent. Hence, failure to identify evidence for small protein function using these methods should not be interpreted as a lack of function.

## CONCLUDING REMARKS

The approaches described above are intended to provide a set of best practices for the detection and validation of small proteins using MS. We also discuss further bioinformatic analysis and prioritization strategies, which are critical for choosing the most promising candidates for subsequent physiological studies. MS approaches can also be used for these functional studies, and provide valuable information on the roles and functions of the novel small proteins. (See Fig. 2 for details [137–142].) For example, MS analyses can be combined with subcellular fractionation to associate small proteins with specific cellular compartments or complexes (81, 104, 141, 142). MS-based proteomics also represent a powerful tool for identification of protein interaction partners for small proteins of interest, when combined with pulldown approaches (for general reviews, see references 121 and 122). A unique advantage of small proteins for these approaches is the ability to chemically synthesize the proteins on a resin, or with modified amino acids that facilitate cross-linking to resin. This obviates the need for epitope tags, which can disrupt protein function. The use of nonnative amino acids can also be incorporated into pulldown methods. A recent study relied on incorporating a nonnative amino acid into small proteins to facilitate cross-linking to interaction partners; while a tag was required to precipitate the small protein, the cross-linking step allowed for the detection of weaker or more transient interactions, as shown for the interaction partners of 24 human small proteins (123). This also highlights the potential of cross-linking mass spectrometry to gain further insights into small protein function. A more detailed description of the options available to elucidate the functions of novel small proteins is provided in the accompanying article of this special issue (124).

In summary, mass spectrometry-based proteomics approaches represent a powerful tool to identify and characterize small proteins. However, the standard workflows available in the field need to be adjusted depending on sample type and complexity, as outlined above. We anticipate that these strategies will provide an excellent starting point for exploratory studies of prokaryotic small proteins.
